# Serotonin 2 Receptors, Agomelatine, and Behavioral and Psychological Symptoms of Dementia in Alzheimer's Disease

**DOI:** 10.1155/2021/5533827

**Published:** 2021-03-31

**Authors:** Hui-Hua Li, Xiao-Yan Yao, Sheng Tao, Xue Sun, Pan-pan Li, Xi-xin Li, Zhu-Li Liu, Chao Ren

**Affiliations:** ^1^Zhenjiang Mental Health Center, The Fifth People's Hospital of Zhenjiang City, Zhenjiang 212000, Jiangsu Province, China; ^2^Department of Neurology, The Affiliated Yantai Yuhuangding Hospital of Qingdao University, Yantai 264000, Shandong Province, China

## Abstract

There are nearly 50 million Alzheimer's disease (AD) patients worldwide, 90% of whom develop behavioral and psychological symptoms of dementia (BPSD), which increase the mortality rate of patients, and impose an economic and care burden on families and society. As a neurotransmitter and neuromodulator, serotonin is involved in the regulation of psychoemotional, sleep, and feeding functions. Accumulating data support the importance of serotonin in the occurrence and development of BPSD. Studies have shown that reduction of serotonin receptors can increase depression and mental symptoms in AD patients. At present, there is no drug treatment for AD approved by the US Food and Drug Administration. Among them, agomelatine, as a new type of antidepressant, can act on serotonin 2 receptors to improve symptoms such as depression and anxiety. At present, research on BPSD is still in the preliminary exploratory stage, and there are still a lot of unknowns. This review summarizes the relationship between serotonin 2 receptors, agomelatine, and BPSD. It provides a new idea for the study of the pathogenesis and treatment of BPSD.

## 1. Introduction

Alzheimer's disease (AD) is a major health burden. In 2016, the International Alzheimer's Disease Association pointed out that there are nearly 50 million AD patients worldwide, 90% of whom develop behavioral and psychological symptoms of dementia (BPSD). It is estimated that by 2050, a new case of AD will occur every 33 s, and nearly 1 million new cases will appear every year. The number of global AD patients may reach 130 million [1, 2], which increases the mortality rate of patients, and imposes an economic and care burden on families and society [3]. AD is ranked fourth in the causes of death among elderly people. The mortality of AD patients with BPSD increases significantly [4]. Considering the safety of patients and others, as well as family members often unable to bear the care and financial burdens, the number of patient visits and hospitalizations is increasing, and even the survival rate is often reduced, which has attracted the attention of medical staff [5, 6]. Therefore, it is urgent to find the pathogenesis and treatment of BPSD. However, a lot of research is still in the preliminary exploratory stage. Genetic studies have suggested that serotonin 2A and 2C genes are likely susceptibility factors for various mental illnesses such as BPSD, schizophrenia, affective disorders, and anxiety [7, 8]. Agomelatine is a new type of antidepressant, a selective agonist of melatonergic MT1 and MT2 receptors and a serotonin 5-HT2C receptor antagonist [9]. Agomelatine has affinity for some members of the serotonin 2 subfamily [10]. In this report, we provide a review of the relationship between serotonin 2 receptors, agomelatine, and BPSD.

## 2. Definition and Epidemiological Characteristics of Alzheimer's Disease (AD) and Behavioral and Psychological Symptoms of Dementia (BPSD)

AD, the most common cause of dementia, is a progressive neurological degenerative disease caused by a variety of biological, psychological, and social factors. Severe memory impairment and cognitive decline may occur, causing severe functional impairment to the patient, as well as imposing a heavy burden on the family and society. A systematic review and meta-regression analysis of dementia in China revealed that the prevalence of AD over the past 30 years (from 1985 to 2015) among people over 55 years of age was 4.03% [11]. Another epidemiological survey showed that in China, the prevalence of dementia among people aged ≥65 years was 5.14%–7.3%, and it is estimated that there are about 8 million dementia patients in China [12]. Some researchers have calculated that the number of dementia patients in China will reach 22 million by 2040. Among them, the proportion with AD is the highest, accounting for 60–70% of all dementias [13].

The clinical manifestations of AD patients include the common cognitive symptoms such as nonrecognition and dyslexia, as well as noncognitive symptoms, which are becoming more important. They are generally called behavioral and psychological symptoms of dementia (BPSD) and are also known as neuropsychiatric symptoms. In 1996, the International Psychogeriatric Association defined BPSD as “a term used to describe a heterogeneous range of psychological reactions, psychiatric symptoms, and behaviors occurring in people with dementia of any etiology” [14]. Burns et al. [15–18] described BPSD in four areas: disorders of thought content (delusions, etc.), disorders of perception (misidentification syndromes, hallucinations, etc.), mood disorders (anxiety, depression, etc.), and behavior disorders (agitation, aggression, etc.). Xue and Zhang [19] summarize BPSD into eight major symptoms: delusions, hallucinations, affective disorders (anxiety, depression, apathy, irritability, etc.), aggressive behavior, abnormal activities, eating disorders, changes in biological rhythms, and sexual dysfunction. These symptoms can occur at any stage of the progression of AD, and the symptoms and frequency can vary. According to epidemiological surveys, BPSD can occur in >90% of AD patients [20]. In the early stage, AD patients may develop reactive depression and anxiety, and in the middle stage, symptoms such as aggression and sleep disturbance often appear. However, in the later stage, due to severe impairment of social functions, depression, emotional apathy, and emotional decline may occur. Studies have found that sleep disorders, irritability, and apathy are considered to be the most common BPSD in patients with AD in China. Among them, apathy is a negative symptom that shows a lack or suppression of passion, emotion, or excitement and is common in AD (70–90%) [21]. Apathy can occur alone or in combination with depression and anxiety. Apathy is one of the most common neuropsychiatric symptoms. Multiple reports indicate that >80% of AD patients have high levels of stress and half have depression and anxiety [22]. Depressive symptoms, as a common type of emotional disorders associated with dementia, have been reported differently. Burns et al. reported a frequency of 0–87%, but most reports are between 14% and 29% [23], and the Chinese literature reports that one-third of BPSD is accompanied by depressive symptoms.

## 3. Serotonin 2 Receptors and Pathogenesis of BPSD

At present, the research on BPSD is mainly based on the summary of clinical characteristics, and there are few reports on its pathogenesis. Therefore, further exploration of the pathogenesis of BPSD is beneficial to the choice of therapeutic drugs and the development of new drugs.

Serotonin, also known as 5-hydroxytryptamine (5-HT), is widely distributed in the central and peripheral nervous systems, mainly in the hippocampus, hypothalamus, and cortex. Forebrain serotonin is derived from dorsal raphe nucleus neurons of the midbrain and participates in the regulation of psychoemotional, sleep, and feeding functions. It is well known that serotonin dysfunction exists in AD patients, which is manifested in the reduction of serotonin and its metabolites in the brain of elderly AD patients, the loss of serotonin neurons in the raphe nucleus, and decreased serotonin 2A receptors in the cortex and hippocampus [24–26]. The reduction of serotonin receptors may be negatively correlated with BPSD [2]. Serotoninergic neurotransmitters are jointly regulated by receptors of different affinity and functionality, as well as serotonin transporters. At present, there are seven serotonin receptor subtypes, and there are different research results in the correlation between each subtype and BPSD. Among them, serotonin 1A, 2A, 2C, 6, and 7 are thought to be related to the occurrence and development of various BPSD [2]. The human serotonin 2A receptor is located on chromosome 13q14-q21 and has high sequence homology with serotonin 2C located at Xq24 [7]. The two receptors are highly conserved between humans, mice, and rats. The reduction of serotonin 2A and 2C receptors and polymorphisms of the genes coding the two receptors are thought to be related to the occurrence of BPSD [26]. Pritchard et al. [7] support a role of 5-HT2A T102C in psychotic symptoms and a role for 5-HT2C cys23ser in anxiety and appetite disturbance. However, the results of human research vary. Some studies have shown that serotonin 2A is associated with an increase in mental illnesses such as depression, while others have shown no relationship and similar findings in serotonin 2C [8, 27, 28]. For example, Japanese researchers have found that Yokukansan (a traditional Japanese herb-medicine) can relieve the head-twitch response (an indicator of hallucination-like symptoms) in the mouse model by mediating downregulation of serotonin 2A receptors in the prefrontal cortex [29]. Shimizu et al. [30] believe that the decrease in serotonin 2A and 2C receptors may be related to the occurrence of BPSD in patients with AD. Holmes et al. [28] found that AD patients with different serotonin 2A and 2C receptor genotypes have a significantly increased risk of depression. However, Pockros et al. [31] believe that the serotonin 2A receptor antagonist and the serotonin 2C receptor agonist can reduce neural excitability and reduce hyperlocomotion, both of which mediate opposing behavioral effects.

**Figure 1:**
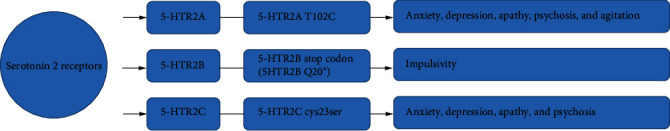
The relationship between 5-HT receptor subtypes and genotypes and BPSD.

Taken together, these studies indicate that the serotoninergic neurotransmitter system is a target for susceptibility to AD and BPSD (Figure 1). The role of these two serotonin 2 receptor subtypes in BPSD needs further study. The relationship between serotonin 2A and 2C receptors, and whether they interact with each other, is an important research topic. This may suggest a new approach to alleviate the symptoms of mental behavior.

## 4. Treatment of BPSD

The treatment of BPSD in AD patients is currently in the exploratory stage. Although BPSD severely affects the quality of life of elderly people with AD and their families, there is no unified and effective drug to control the progress of AD cognitive impairment. However, compared with the cognitive dysfunction of AD, we can better control BPSD through appropriate psychiatric intervention and treatment, improving patient symptoms and quality of life, and reducing the burden on caregivers.

At present, there are two main types of therapeutic schedules for BPSD: nonpharmacological and pharmacological interventions. Chinese guidelines for the diagnosis and treatment of dementia and cognitive impairment recommend nonpharmacological treatments as a first-line treatment for BPSD, including music therapy, cognitive behavioral therapy, physical therapy, etc. The memory therapy developed by Butleras has been reported to improve depressive symptoms in patients with BPSD [32], but its onset is often slower and is suitable for patients with mild disease. Therefore, in patients in whom nondrug treatment is not effective, drug intervention is needed. At present, the drug treatment of AD generally uses nootropic drugs. Acetylcholinesterase (ACHE) inhibitors (e.g., donepezil) and N-methyl-D-aspartic acid (NMDA) receptor antagonists (such as memantine) are representative [33]. For severe BPSD, additional psychotropic drugs are needed, including antidepressants, antipsychotics, anxiolytics, and sedative hypnotics.

With the deepening of research, it has been found that antidepressants may also play a role in the treatment of AD and BPSD. Although some antidepressants have been applied to adjuvant treatment of AD and BPSD in clinical practice and proved to be effective, the effectiveness of various antidepressants is not the same. Based on evidence-based medicine and the National Institute of Health and Clinical Optimization (NICE) clinical treatment guidelines, Jeyapaul et al. proposed that for the treatment of BPSD in AD patients, serotonin reuptake inhibitors (SSRIs) are preferred for depression. Sertraline or citalopram are the preferred SSRIs [34]. Studies have mostly used citalopram for research. It is reported that taking 30 mg/day citalopram has a positive effect on agitation in dementia. Unfortunately, this study also confirmed the risk of causing QT extension [35]. Tricyclic and tetracyclic antidepressants have many adverse reactions and should be used with caution in elderly patients. Venlafaxine is an antidepressant in the class of selective serotonin-norepinephrine reuptake inhibitors. It has a quick onset and few adverse reactions, so it can be used as appropriate. Studies have shown that new antidepressants may be most helpful in the treatment of restlessness, while depression, apathy, and anxiety are worse in dementia [33, 36]. As a new antidepressant, the application of agomelatine in AD and BPSD is worth exploring. Some experiments show that agomelatine have an anti-AD effect through repairing cholinergic system dysfunction, serotonergic system dysfunction, and glutamate/GABA system dysfunction; eliminating the *β*-amyloid protein; and inhibiting oxidative stress on multiple targets and multiple pathways. And there is also evidence that agomelatine has a unique pharmacological effect and improves depression, depressive symptoms, and sleep cycles in patients with AD [37]. Agomelatine can improve apathy in frontotemporal dementia [38]. Yao et al. [37] presented that agomelatine could prevent A*β*-induced tau phosphorylation and oxidative damage in PC12 cells; agomelatine exerts neuroprotection in AD. So, its application in BPSD is worthy of clinical attention in the future.

Clinical studies have shown that the use of antipsychotic drugs can reduce BPSD, especially second-generation antipsychotics (SGAs) such as clozapine, risperidone, olanzapine, and quetiapine. SGAs work by blocking the serotonin receptor and dopamine receptor pathways and alleviating delusions, hallucinations, agitation, and aggressive behavior. Risperidone is approved for the treatment of AD. However, most literature also points out that there are safety issues in the treatment of BPSD with antipsychotic drugs, which have increased adverse events such as long-term tardive dyskinesia, cardiovascular and cerebrovascular accidents, and liver and kidney damage in AD patients.

As a result, the US Food and Drug Administration issued a black box warning that the use of antipsychotic drugs in patients with dementia increases the risk of death. The use of benzodiazepines and mood stabilizers to treat BPSD in patients with dementia is currently not recommended. This is because published data indicate a high risk of adverse reactions and a low probability of effective treatment [39].

## 5. Possible Mechanism of Interaction between Agomelatine, Serotonin, and BPSD

Treatment of AD is mainly to delay disease progression, and treatment of BPSD is to improve symptoms and reduce the burden on caregivers. At present, the treatment of BPSD has become an important medical challenge for elderly patients. The two major pathological features of AD are the deposition of extracellular amyloid-beta (A*β*) peptides in the brain, leading to the formation of amyloid plaques and excessive phosphorylation of Tau protein to form neurofibrillary tangles. Studies have found that serotonin receptors affect the two major pathological features of AD. For example, AD mouse models that overexpress A*β* indicate that the pathological accumulation of A*β* depends on the reduction of serotonin 2A receptor expression [40]. Antagonizing 5-HTR2C can prevent tau hyperphosphorylation and enhance memory [41]. Studies have shown that melatonin and indole compounds (mainly serotonin) can inhibit and destabilize A*β* fibril formation, increase cell viability by 9–25%, and have neuroprotective effects [42]. The serotonin 2 receptor family has become the developmental target of pharmacological treatments for a variety of mental disorders, including depression, schizophrenia, and addiction.

The new-generation antidepressant agomelatine has unique pharmacological properties and mechanism of action. Most studies have shown that agomelatine is a postsynaptic serotonin 2C receptor antagonist and melatonin receptor agonist [9]. It acts on the suprachiasmatic nucleus, hippocampus, frontal cortex, and striatum, leading to improved sleep duration, recovery of circadian rhythm, and improved mood. It has a positive effect on anxiety and depression. However, the drug does not adversely affect memory and attention during treatment, especially in elderly patients; does not affect blood pressure and heart rate; and does not cause habitual and withdrawal symptoms and behavioral toxicity [43]. At the same time, it can increase noradrenergic and dopaminergic neurotransmissions [44]. The serotonin is also a precursor of pineal melatonin; Zhou et al. found that melatonin was significantly reduced in cerebrospinal fluid samples from AD. In order to find new drugs for the treatment of AD, a large-scale study that analyzes and predicts the global database of AD treatment drugs showed that agomelatine can interact with nine AD-related targets (serotonin 2A receptor, ADORA2A, ACHE, BACE1, PTGS2, GABRG1, MAOB, SIGMAR1, and ESR1) to treat AD by repairing the serotonin, GABA, and cholinergic system [45]. This is different from the previous thought that agomelatine acts on the serotonin 2C receptor. Agomelatine could improve apathy and depression [33]. In 2016, researchers reported a case report. A 91-year-old AD patient with sleep disorders and depression symptoms improved significantly after using agomelatine [46]. Treatment of insomnia and depression in AD patients also has a positive effect on cognitive function. It provides a new direction for us to study the treatment of BPSD in AD patients. However, there are some reports that the use of drugs such as SSRIs and agomelatine can cause emotional deactivation in AD patients. Some researchers believe that this emotional disorder induced by AD is due to dopamine neurotransmission in the neural circuit that regulates reward processing downregulation and occurs secondary to the activation of serotonin 2C receptors in the nucleus accumbens [47].

However, the relationship between agomelatine and serotonin receptor and AD has been proven inconclusively. It is not clear what the specific relationship between agomelatine, serotonin receptor, and BPSD is. There are few reports on the relationship between the efficacy of agomelatine in the treatment of BPSD and serotonin receptors (especially considering both serotonin 2A and 2C receptors). No significant changes in the two receptors following agomelatine have been reported.

## 6. Discussion and Conclusion

**Figure 2:**
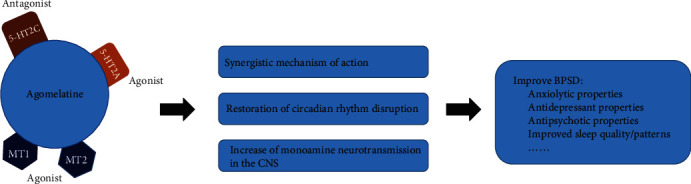
The possible mechanism of agomelatine to improve BPSD.

With the aging of society, the prevalence of AD is increasing rapidly. About 90% of people with dementia have at least one BPSD. These symptoms include depression, anxiety, and apathy. The improvement of these symptoms helps to improve the quality of life of patients. Therefore, it is urgent to find the pathogenesis and treatment of BPSD. However, the treatment of BPSD is currently in the preliminary exploratory stage. Many research results are also conflicting. This review attempts to find associations between serotonin, agomelatine, and BPSD. We put forward the following hypothesis: agomelatine can improve BPSD in AD patients and mainly works by acting on serotonin 2A and 2C receptors, causing the serotonin concentration in the brain to increase and have changes in other monoamine transmitters. Thereby, it can further change mental symptoms, such as anxiety and depression (Figure 2). However, BPSD have a complex mechanism. This hypothesis also needs to be confirmed. The optimal drug therapy for BPSD is still under exploration. Antidepressants are currently being given high hopes, but the effects of antidepressants on BPSD vary considerably. Among them, agomelatine is a noteworthy candidate; accordingly, it can improve apathy, depression, anxiety, insomnia, etc. in BPSD. However, we should also be aware that everything has two sides; agomelatine also has its shortcomings. For instance, agomelatine has less evidence for efficacy in elderly patients, and the risk of liver damage may increase. This review is aimed at providing a new idea for the study of the pathogenesis and treatment of BPSD. We hope that in the future, we can clarify (1) what serotonin changes exist in patients with BPSD, (2) whether serotonin changes cause BPSD or become a marker for detecting BPSD, and (3) the link between BPSD and serotonin receptor subtypes.
